# A Double-Blind Randomized Controlled Trial of Maternal Postpartum Deworming to Improve Infant Weight Gain in the Peruvian Amazon

**DOI:** 10.1371/journal.pntd.0005098

**Published:** 2017-01-05

**Authors:** Layla S. Mofid, Martín Casapía, Eder Aguilar, Hermánn Silva, Antonio Montresor, Elham Rahme, William D. Fraser, Grace S. Marquis, Jozef Vercruysse, Lindsay H. Allen, Brittany Blouin, Hugo Razuri, Lidsky Pezo, Theresa W. Gyorkos

**Affiliations:** 1 Department of Epidemiology, Biostatistics and Occupational Health, McGill University, Montréal, Québec, Canada; 2 Division of Clinical Epidemiology, Research Institute of the McGill University Health Centre, Montréal, Québec, Canada; 3 Asociación Civil Selva Amazónica, Iquitos, Peru; 4 Hospital Iquitos "César Garayar García", Iquitos, Peru; 5 Department of Control of Neglected Tropical Diseases, World Health Organization, Geneva, Switzerland; 6 Department of Medicine, McGill University, Montréal, Québec, Canada; 7 Centre de recherche et Département d’obstétrique et de gynécologie, Université de Sherbrooke, Sherbrooke, Québec, Canada; 8 School of Dietetics and Human Nutrition, McGill University, Ste. Anne-de-Bellevue, Québec, Canada; 9 Department of Virology, Parasitology and Immunology, Faculty of Veterinary Medicine, Ghent University, Merelbeke, Belgium; 10 USDA, ARS Western Human Nutrition Research Center, University of California, Davis, California, United States of America; University of Kelaniya, SRI LANKA

## Abstract

**Background:**

Nutritional interventions targeting the critical growth and development period before two years of age can have the greatest impact on health trajectories over the life course. Compelling evidence has demonstrated that interventions investing in maternal health in the first 1000 days of life are beneficial for both mothers and their children. One such potential intervention is deworming integrated into maternal postpartum care in areas where soil-transmitted helminth (STH) infections are endemic.

**Methodology/Principal Findings:**

From February to August 2014, 1010 mother-infant pairs were recruited into a trial aimed at assessing the effectiveness of maternal postpartum deworming on infant and maternal health outcomes. Following delivery, mothers were randomly assigned to receive either single-dose 400 mg albendazole or placebo. Participants were followed-up at 1 and 6 months postpartum. There was no statistically significant difference in mean weight gain between infants in the experimental and control groups (mean difference: -0.02; 95% CI: -0.1, 0.08) at 6 months of age. Further, deworming had no effect on measured infant morbidity indicators. However, *ad hoc* analyses restricted to mothers who tested positive for STHs at baseline suggest that infants of mothers in the experimental group had greater mean length gain in cm (mean difference: 0.8; 95% CI: 0.1, 1.4) and length-for-age z-score (mean difference: 0.5; 95% CI: 0.2, 0.8) at 6 months of age.

**Conclusions/Significance:**

In a study population composed of both STH-infected and uninfected mothers, maternal postpartum deworming was insufficient to impact infant growth and morbidity indicators up to 6 months postpartum. Among STH-infected mothers, however, important improvements in infant length gain and length-for-age were observed. The benefits of maternal postpartum deworming should be further investigated in study populations having higher overall prevalences and intensities of STH infections and, in particular, where whipworm and hookworm infections are of public health concern.

**Trial registration:**

ClinicalTrials.gov (NCT01748929).

## Author Summary

Worldwide, over one billion people are infected with intestinal worms (roundworms, whipworms, and hookworms). In worm-endemic areas, women of reproductive age are a high risk group for infection because of their poor nutritional status and increased physiological needs during pre-pregnancy, pregnancy, and lactation. To measure the effect of providing mothers with deworming treatment soon after delivery, we conducted a trial in 1010 mother-infant pairs. Mothers were randomly assigned to receive either a single-dose deworming tablet or a placebo tablet. Mothers and their infants were visited in their homes at 1 and 6 months following delivery. At the 6-month time point, among all mother-infant pairs, we could not detect an effect of deworming on infant growth or morbidity. We did, however, observe that, among women who were infected with intestinal worms at baseline, deworming had a beneficial effect on important infant growth outcomes. The potential benefit of maternal postpartum deworming in populations with higher prevalences and intensities of intestinal worms, particularly where infections with whipworm and hookworm predominate, warrants further investigation.

## Introduction

Worldwide, more than 200 million children under five years of age fail to reach their full developmental potential [1]. Suboptimal nutrition and growth during fetal and early childhood development are widely regarded as having adverse long-term consequences, including higher susceptibility to infections, decreased learning potential, lower economic productivity, and greater risk of mortality [2]. The most critical period of pre- and postnatal development (coined the first 1000 days of life) is a time when interventions can have the greatest impact on preventing negative health and economic outcomes over the life course [3, 4]. Given the intrinsic relationship between maternal and child health, the nutritional status of mothers is considered to be a pivotal driver of infant growth, development, and survival [5, 6]. To date, most studies have focused almost exclusively on maternal micronutrient deficiencies [7] and have not adequately addressed the multifactorial causes of maternal malnutrition, including the role of maternal infection in future infant growth and morbidity.

The soil-transmitted helminth (STH) infections (*Ascaris lumbricoides* (the roundworm), *Trichuris trichiura* (the whipworm) and the hookworms, *Necator americaus* and *Ancylostoma duodenale*) are considered the most prevalent parasitic infections of humans, affecting more than 1.45 billion people worldwide [8]. The global significance of STHs lies in their high burden of disease, attributed to blood loss, anemia, and malabsorption of nutrients that can cause or further exacerbate nutritional deficiencies [9]. Sequelae of chronic infection with STHs include fatigue, loss of appetite, growth faltering, and impaired cognition [9–11]. While much of the current research and focus of large-scale control programs have targeted preschool and school-aged children, women of reproductive age (WRA) remain a largely neglected group [12] despite their underlying poor iron and micronutrient status due to inadequate diet and high risk of blood loss due to menstruation, pregnancy, and childbirth [11, 13, 14]. While species-specific estimates are not available for WRA as a whole, it is estimated that, at any one time, as many as 37.7 million WRA are infected with hookworms alone [15].

The World Health Organization (WHO) recommends that single-dose deworming (i.e., single tablets of 400 mg albendazole or 500 mg mebendazole) be given to WRA, including pregnant women (after the first trimester) and lactating women, in endemic areas where the prevalence of infection exceeds 20% [14]. Among the 112 countries considered endemic for STH infections [16], no coverage estimates are available for deworming in WRA (i.e., the proportion of WRA reached by the deworming intervention), and few countries include this risk group among other deworming activities [17]. The evidence base on deworming in WRA is limited, and largely focuses on deworming during pregnancy. Randomized controlled trials (RCTs) and observational studies conducted in pregnant women have been largely inconclusive, providing mixed evidence on the benefits of deworming on maternal anemia, low birthweight, and perinatal mortality [15, 18, 19]. The uptake and health benefits of deworming during pregnancy may not be optimal because in many low-and-middle-income countries antenatal care attendance is low and reinfection rates can be high. The early postpartum period presents an innovative opportunity to reach WRA because deworming can be easily integrated into routine care, and many low-and-middle-income countries have, or are currently adopting and actively promoting, pro-hospital and health centre delivery policies.

While maternal deworming will be of direct benefit to the infected woman (by curing or reducing her burden of STH infection), the unique interface between mother and child during lactation suggests that benefits may also accrue to the newborn infant. To address this research gap, we designed an RCT aimed at comparing the effectiveness of single-dose albendazole *vs*. placebo administered to women following hospital delivery on infant growth and morbidity in an area of Peru known to be highly endemic for STH infection.

## Methods

### Research design and setting

This study is a randomized, double-blind, placebo-controlled trial conducted in the Amazon region of Iquitos, Peru. A recent meta-analysis estimated that a quarter of the population in Peru was infected with STHs between 2005 and 2012 [20]. In Iquitos, the prevalence of infection has been reported to be much higher than the country average with prevalences > 80% in school-aged children [21], > 40% in preschool-aged children [22], and > 90% in pregnant women [23]. Iquitos was chosen as the study site because of its high STH endemicity, a high proportion of women delivering in hospital, and because, at the time of the trial, there were no routine deworming programs targeting WRA.

### Recruitment

A detailed description of study procedures can be found in the trial protocol [24]. Briefly, beginning in February 2014, mother-infant pairs were enrolled into the trial using a two-stage approach. First, from pregnancy registries accessed in health centre records, women who were in their third trimester of pregnancy were visited in their homes by research personnel in order to assess eligibility using the following inclusion criteria: planned delivery at Hospital Iquitos “César Garayar García”; and intention to remain in the study area for a 24-month period. Women were ineligible to participate in the trial based on the following exclusion criteria: confirmed pregnancy of multiples; and inability to communicate in Spanish. Following a detailed explanation of the trial, women and their partners were asked to provide informed consent and, if this was obtained, a baseline questionnaire was administered.

Upon arrival at the Hospital Iquitos “César Garayar García” for delivery, women were approached by research assistants and asked to re-confirm their consent to participate in the trial. Following delivery, mothers and newborns were assessed for the following exclusion criteria: stillborn; serious congenital abnormality or serious medical condition (e.g., exposure to HIV infection); gestational age < 32 weeks; Apgar score < 4 at 5 minutes (Apgar score is a scored evaluation of the physical condition of an infant immediately following delivery. The infant's heart rate, respiratory effort, muscle tone, reflex irritability, and color are assessed by clinical personnel [25]. Each component is scored from 0 to 2 and then the scores are summed, giving a total score between 0 and 10.); mother or baby transferred to another hospital; and hospitalization of mother or baby for > 3 days. In the case that women presented at the hospital for delivery prior to being visited in their home by a research assistant, every effort was made to recruit mothers into the trial as soon as possible before or after delivery (i.e., within 10 hours of birth).

### Randomization

A computer-generated randomization schedule was prepared prior to recruitment by a statistician not otherwise involved in the trial, using a random number sequence according to simple randomization with a 1:1 allocation ratio. Group assignments were concealed in opaque sequentially-numbered envelopes according to the randomization schedule. Envelopes were stored in a secure temperature-regulated pharmacy. A print copy of the randomization schedule was stored in a sealed envelope under lock-and-key at the research office in Canada (Research Institute of the McGill University Health Centre).

### Deviation from protocol

Initially, the randomization sequence had been planned as a permuted block design with randomly varying block sizes of 6 and 8 with a 1:1 allocation ratio. Due to a miscommunication, the statistician prepared a simple randomization sequence with a 1:1 allocation ratio. With a large sample size and a similar rate of recruitment over time into the two intervention groups, either randomization sequence would be expected to produce balanced intervention groups, as was the case in this trial (see Table 1).

**Table 1:** Baseline characteristics of mothers and infants (N = 1010) by intervention group, Iquitos, Peru (February–August 2014).

	Albendazole (n = 510)	Placebo (n = 500)
***Maternal characteristics***		
Age (years) mean ±SD	25.2 ±6.8	25.6 ±7.0
Married or cohabiting *n* (%)	447 (87.7)	443 (88.6)
Primigravida *n* (%)	134 (26.3)	127 (25.4)
Less than secondary education *n* (%)	291 (57.1)	299 (59.8)
Employment outside home *n* (%)	60 (11.8)	58 (11.6)
Deworming during pregnancy *n* (%)	11 (2.2)	8 (1.6)
Iron supplementation during pregnancy *n* (%)	478 (93.7)	477 (95.4)
Vaginal delivery *n* (%)	393 (77.1)	378 (75.6)
***Infant characteristics***		
Male *n* (%)	248 (48.6)	251 (50.2)
Birthweight (kg) mean ±SD	3.2 ±0.4	3.2 ±0.4
Birth length (cm) mean ±SD	48.6 ±1.8	48.6 ±1.9
Birth head circumference (cm) mean ±SD	33.6 ±1.3	33.6 ±1.2
Gestational age (weeks) mean ±SD	38.7±0.8	38.6 ±0.9
Apgar score at 5 minutes mean ±SD	9.8±0.5	9.8 ±0.6
***Household characteristics***		
Peri-urban or rural residence *n* (%)	490 (96.1)	485 (97.0)
Access to potable water in home *n* (%)	396 (77.7)	367 (73.4)
Home with dirt or wooden floor *n* (%)	321 (62.9)	352 (70.4)
Number of people residing in household mean ±SD	5.9 ±2.8	5.8 ±2.6

SD = standard deviation

### Treatment allocation and blinding

Eligible, consenting women were visited at bedside prior to hospital discharge and were administered the tablet in the next sequentially-numbered envelope. Participants, research personnel, and data analysts were blinded to group assignment. Mothers randomized to the experimental group received single-dose 400 mg albendazole and mothers randomized to the control group received single-dose placebo. The albendazole tablets were manufactured by GlaxoSmithKline Inc. and donated for this trial by WHO. The placebo tablets were manufactured to be identical to the albendazole tablets in all aspects, including size, taste, color, and markings and were manufactured by Laboratorios Hersil in Lima, Peru. Both groups received the current standard of routine postpartum care from hospital personnel. At 6 months postpartum, all mothers were offered deworming as part of the trial protocol, if they were not newly pregnant (i.e., in the first trimester of pregnancy).

### Sample size

The required sample size was estimated for the primary outcome of mean infant weight gain between birth and 6 months of age. An estimate of mean weight gain was obtained from recent data on children between 5 and 7 months of age residing in the study area (i.e., 4.24 kg with a standard deviation of 1.014 kg) [26]. The trial sample size was computed using a two-sided independent *t*-test, with a significance level of 0.05, a power of 0.80, a standard deviation of 1.014 kg, a minimum detectable difference in weight gain of 0.2 kg, and taking into account an attrition rate of 20%. Based on the above specifications, a total of 1010 participants was calculated to be the sample size needed to declare that intervention groups would be different if, in fact, there would be a true between-group difference of 0.2 kg or more in weight gain between birth and 6 months of age. Sample size calculations were carried out using PS Power and Sample Size Calculations version 3.0 (Dupont and Plummer, 2009). Based on the number of deliveries at the study hospital in 2011, recruitment was expected at the rate of 300 mother-infant pairs per month, suggesting a total recruitment period of approximately 3.5 months.

### Baseline assessment

#### Sociodemographic and clinical information

A baseline questionnaire was administered to women following receipt of informed consent to obtain information on sociodemographic characteristics. Pregnancy and delivery information about mothers (e.g., pregnancy complications) and their infants (e.g., gestational age, Apgar score, and birth time) was obtained from medical charts prior to randomization.

#### Baseline soil-transmitted helminth infection

The analysis of fresh stool specimens using the Kato-Katz technique is recommended for the assessment of STH prevalence and also to quantify the intensity of infection [27]. While specificity for a single stool specimen using this technique has been estimated at over 93%, sensitivity has been reported at 96.9% (95% Bayesian Credible Interval [BCI]: 96.1, 97.6) for *A*. *lumbricoides*, 91.4% (95% BCI: 90.5%, 92.3%) for *T*. *trichiura*, and 65.2% (95% BCI: 60.0%, 69.8%) for hookworm [28].

Baseline assessment of STH infection in the total trial population was not possible because of ethical constraints preventing the diagnosis of STH infection and subsequent randomization to a placebo control group. In order to estimate an accurate baseline prevalence and intensity of STH infection, women were asked to provide a stool specimen in the hospital when they presented for delivery. Only specimens collected from participants randomized to the experimental group were analyzed immediately using the Kato-Katz technique. This is because they would have received deworming treatment as part of the trial protocol, therefore respecting ethical guidelines. Due to randomization, it was expected that the baseline STH infection in the experimental group would be similar to that in the control group, thereby providing an accurate STH infection profile of the trial population at baseline. Blinding of laboratory technologists, research personnel, and study coordinators was preserved using a code-switching method that had been successfully implemented in another trial [29]. Briefly, the local study coordinator replaced participant identification codes on specimen containers with a laboratory code prior to transfer to the laboratory, according to a pre-defined list. In the laboratory, technologists were provided another list containing laboratory codes and indications of which specimens were to be analyzed immediately and which were to be stored. Each list was kept on a password-protected computer, one in the administration office of the trial in Peru (Asociación Civil Selva Amazónica), and the other in the laboratory. A master list linking information was kept under lock-and-key at the research office in Canada (Research Institute of the McGill University Health Centre).

As uninfected individuals are not expected to benefit from deworming treatment, experts have argued that deworming trials should restrict data analyses to those participants who test positive for STH infection at baseline, so as to best estimate the effectiveness of treatment [30]. In order to obtain a baseline measure of STH infection in the total trial population, all women were asked to provide a stool specimen during the first stage of recruitment (i.e., in participants’ homes during pregnancy). All specimens collected at this time point were stored in 10% formalin and analyzed by the direct smear and ethyl-ether concentration techniques after the 6-month study visit when all women received deworming as part of the trial protocol. Sensitivity for the direct smear technique has been estimated at 52.1% (95% BCI: 46.6%, 57.7%), 62.8% (95% BCI: 56.9%, 68.9%), and 42.8% (95% BCI: 38.3%, 48.4%) for *A*. *lumbricoides*, *T*. *trichiura* and hookworm, respectively [31]. Sensitivity for fecal concentration techniques have been estimated at 56.9% (95% BCI: 51.1%, 63.5%) for *A*. *lumbricoides*, 81.2% (95% BCI: 73.0%, 89.2%) for *T*. *trichiura*, and 53.0% (95% BCI: 48.6%, 57.5%) for hookworm [31].

### Outcome ascertainment

The pre-specified primary outcome measure was mean infant weight gain between birth and 6 months of age. Pre-specified secondary infant outcome measures were length and head circumference (HC) gains, derived growth indices (i.e., weight-for-age (WAZ), weight-for-length (WFL), length-for-age (LAZ), HC-for-age (HCAZ), and MUAC-for-age (ACAZ)), prevalence of underweight, wasting and stunting, and occurrence of infant morbidity (i.e., occurrence of hospitalizations since birth, and incidence of diarrhea, respiratory problems, fever, and ear infections) at 1 and 6 months postpartum. Secondary maternal health outcomes (i.e., STH infection and intensity, anemia, fatigue), and breast milk outcomes (i.e., milk quality and quantity) are reported separately. This article presents infant health outcomes up to 6 months postpartum (at which time the primary outcome was measured). However, follow-up for the trial will continue until the 24-month time point.

#### Anthropometry

Anthropometry in infants was measured immediately after delivery in the hospital, and during follow-up, at participants’ homes at 1 and 6 months following delivery. Weight was measured in the unclothed infant using a digital balance (Seca 354, Seca Corp., Baltimore, USA), accurate to 0.01 kg. Recumbent length was measured using a stadiometer (Seca 417, Seca Corp., Baltimore, USA), accurate to 0.1 cm. HC was measured using non-stretchable tape (Seca 212, Seca Corp., Baltimore, USA), accurate to 0.1 cm. Mid-upper arm circumference (MUAC) was measured at the 6-month study visit only using non-stretchable tape (UNICEF S0145620), accurate to 0.1 cm. All anthropometric measurements were performed in duplicate and the mean value was used for analyses. All research assistants were trained on anthropometry assessment according to WHO guidelines [32] in order to ensure accuracy, precision, and standardization of measurements.

#### Infant morbidity

At each follow-up visit, information about infant morbidity and hospitalization since birth was obtained by self-report from the mothers. Infant morbidity indicators were adapted from the Integrated Management of Childhood Illness (IMCI) [33] and included episodes of diarrhea, cough or difficulty breathing, fever, and ear infections in the previous two weeks. In the case of reported hospitalizations since birth, hospital medical charts were reviewed to confirm admission, diagnosis, and treatment. Information on serious adverse events (SAEs) was obtained through passive reporting of mothers at study visits or between study visits. All SAEs were categorized according to WHO guidelines [14] and reported to the relevant research ethics boards and the trial’s Data Safety and Monitoring Committee (DSMC).

Every attempt was made to visit trial participants as close to the target date of their scheduled study visit as possible in order to ascertain the most accurate anthropometric data. To minimize loss to follow-up, research assistants made numerous attempts to locate and/or communicate with participants. Participants were considered lost to follow-up only in circumstances where they could not be located; the area of relocation was too far away; either the mother or child had died; or due to voluntary withdrawal from the study. All data collected during home visits were recorded electronically using a data application designed specifically for the trial (i.e., e-EPI) on a mobile tablet and reviewed daily for accuracy and completeness by the research coordinator (LP) and project director (LSM).

### Statistical analyses

#### Derived variables

Weight, length and HC gains were calculated by subtracting the values at each study visit by the values at baseline. Growth indices (i.e., WAZ, WFL, LAZ, HCAZ, ACAZ) were calculated using Anthro software version 3.2.2 (WHO, 2011) and macros for Stata. The prevalences of underweight, wasting, and stunting were defined as z-scores for WAZ, WFL and LAZ respectively, of < -2 SD from the median of the WHO reference population.

#### Baseline soil-transmitted helminth infection

For the analysis of fresh stool specimens using the Kato-Katz technique, Bayesian models were used to correct prevalence estimates by adjusting for imperfect sensitivity and specificity in the absence of a gold standard, for each helminth species. An uninformative prior (i.e., beta distribution with the parameters α = 1, β = 1) was used for the baseline prevalence and prior information on sensitivity and specificity for each helminth species were taken from a recent publication by Tarafder *et al*. (2010) [28]. Bayesian analyses were performed using the BayesDiagnosticTests software package version 3.10.2 (2016), using the methods of Joseph *et al*. (1995) [34]. For the analysis of stored stool specimens, participants were classified as infected if testing positive on any one of the two techniques (i.e., direct smear and ethyl-ether concentration), and uninfected if testing negative on both techniques.

#### Effect of deworming on primary and secondary infant outcomes

The effectiveness of deworming on infant growth and morbidity outcomes was first assessed according to the intention-to-treat (ITT) principle, such that participants were analyzed according to their assigned intervention group. Multiple Imputation by Chained Equations (MICE) with 20 imputations was used to impute anthropometric and morbidity outcome data for those infants who missed their 1 and/or 6-month follow-up visits. Variables that were related to the outcome, and/or related to loss-to-follow-up were included in the imputation model, including weight, length and HC at birth, infant sex, gestational age, maternal age, marital status, education level, number of people residing in the household, socioeconomic status, and intervention group.

As one objective of the trial was to assess the possibility of effect measure modification by infant sex, birthweight and birth length on infant growth indices, the initial imputation model also included interaction terms for intervention group and infant sex, birthweight, and birth length. However, since no evidence of interaction was observed in the analysis models, interaction terms were dropped from the final imputation model.

Unadjusted ITT analyses for continuous outcomes were performed using univariable linear regression. Unadjusted ITT analyses for dichotomous outcomes were performed using univariable log-binomial regression modeling. Participants were categorized into quartiles of socioeconomic status using an asset-based index derived from the following six variables: presence of a gas stove, television, radio, electricity in the house, tap water connection in the house, and housing material of cement or bricks [29, 35, 36]. The index was constructed using weights derived from Principal Components Analysis. Adjusted analyses used multivariable linear regression modeling for continuous outcomes and multivariable log-binomial regression for dichotomous outcomes, with the following covariables: maternal age, education, socioeconomic index, infant sex, and gestational age. In the case where log-binomial regression models could not converge, the risk ratio was estimated using a Poisson regression model with a robust variance estimator [37, 38].

The following sensitivity analyses were also planned, *a priori*, to be undertaken using: 1) a complete-case approach which included all participants who had complete outcome data for the 1 and 6-month follow-up visits; and 2) a per-protocol approach which included those who had complete outcome data for the 1 and 6-month follow-up visits and did not report taking deworming outside of the trial protocol at either time point. In the literature, meta-analyses of deworming trials commonly conduct analyses in datasets in which deworming was administered to both infected and uninfected individuals, and in datasets restricted to only STH-infected individuals [39]. Therefore, we also performed analyses restricted to STH-infected mothers at baseline. All statistical analyses were carried out using Stata/SE version 14.0 (StataCorp, College Station, Texas, 2015).

### Ethical considerations

Ethics approval for this trial was obtained from the research ethics committees of the Asociación Civil Impacta Salud y Educación (Peru), the Instituto Nacional de Salud (Peru) and the McGill University Health Centre (Canada). As per Peruvian Institute of Health guidelines, all women and their partners provided written informed consent for participation in the trial. In the case that a woman or her partner was under the age of 18 years, written assent was obtained, and informed consent was obtained from their parent, guardian or spouse/partner over the age of 18 years. Women who did not have a partner, or whose partner was absent for an indefinite period of time (e.g., death, separation, etc.), were asked to sign a sworn statement to declare the father’s absence, in accordance with Peruvian ethics guidelines.

An independent DSMC comprised of three international experts was put in place to monitor trial progression and ensure participant safety. At pre-specified time points (i.e., after 50% and 100% of recruitment, and after the completion of the 1 and 6-month study visits), the DSMC reviewed all SAEs and approved trial continuation. The trial was registered prior to commencement with ClinicalTrials.gov (NCT01748929), and the trial protocol is available as a published manuscript [24]. The Consolidated Standards of Reporting Trials (CONSORT) checklist can be found in S1 Checklist.

## Results

### Trial recruitment

**Fig 1:**
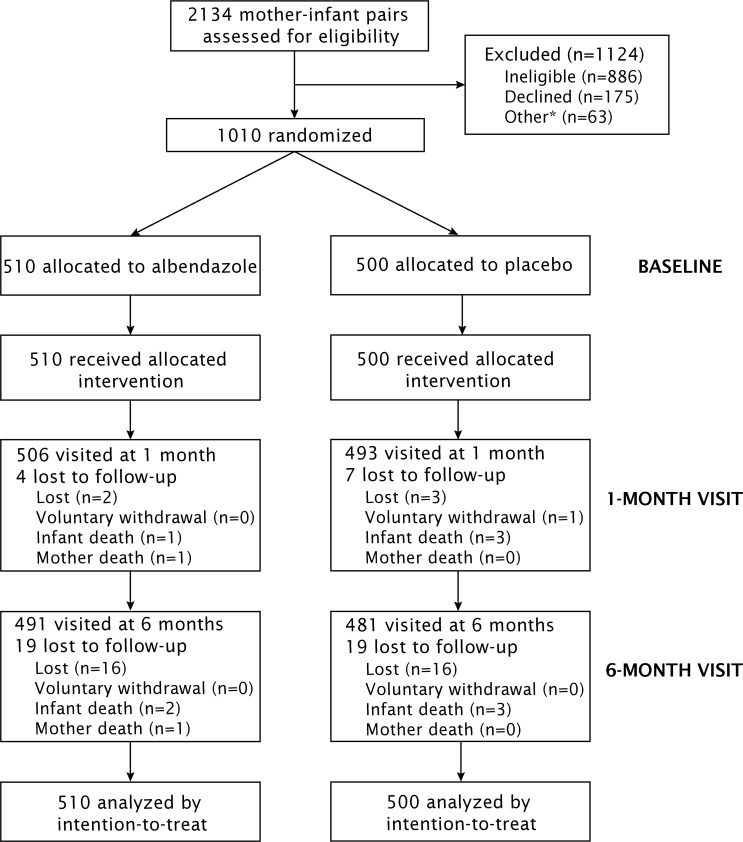
CONSORT trial flow diagram. *63 women were pre-recruited but were not enrolled because the sample size was already met. It should be noted that some participants who were lost to follow-up at the 1-month visit were recuperated at the 6-month visit.

Recruitment into the trial took place between February and August 2014. Of the 2134 women and their partners who were approached to participate in the trial, 1010 were enrolled and randomized (Fig 1). The remaining 1124 mother-infant pairs did not participate because: the mother did not meet the pre-delivery eligibility criteria (n = 752), either mother or infant did not meet the post-delivery eligibility criteria (n = 134), either mother or her partner declined to participate (n = 175), or the sample size was reached before enrollment could take place (n = 63). Those who declined to participate were more likely to live in urban areas (OR: 2.8; 95% CI: 1.4, 5.5), and be primigravida (OR: 1.6; 95% CI: 1.1, 2.4). All 1010 mother-infant pairs enrolled into the trial were randomized to the experimental (n = 510) or control (n = 500) groups.

### Description of study population

Baseline maternal and infant characteristics for the two intervention groups are summarized in Table 1. Groups were similar in terms of maternal and infant characteristics. However, some differences between groups were found in household characteristics (i.e., access to potable water in the home and the type of housing structure).

In the hospital, 64 participants (12.5%) in the experimental group provided a stool specimen that was analyzed immediately using the Kato-Katz technique. Those who provided a stool specimen for analysis in the hospital were similar in terms of baseline characteristics to those who did not provide a specimen. Due to the RCT design, prevalences and intensities of infection were expected to be similar between intervention groups. Uncorrected and corrected prevalences of STH infection, along with intensities are presented in Table 2. The proportion of women testing positive for any STH infection was 48.4% (31/64), of which 29.7% (19/64) tested positive for *A*. *lumbricoides*, 26.6% (17/64) tested positive for *T*. *trichiura*, and 6.3% (4/64) tested positive for hookworm. Only 7 women had co-infections: 2 with all 3 species and 5 with *A*. *lumbricoides* and *T*. *trichiura*. Bayesian methods that adjusted for imperfect sensitivity and specificity produced corrected prevalences of 31.4% (95% BCI: 13.7%, 49.4%) for *A*. *lumbricoides*, 18.5% (95% BCI: 1.0%, 43.5%) for *T*. *trichiura*, and 28.7% (95% BCI: 1.9%, 88.6%) for hookworm. The mean eggs per gram (epg) values for each helminth species were of low intensity according to WHO thresholds [40].

**Table 2:** Baseline prevalence and intensity of soil-transmitted helminth infections as assessed by single stool specimens collected in the hospital (n = 64) and analyzed using the Kato-Katz technique, Iquitos, Peru (February–August 2014)

STH species	Prevalence
***A*. *lumbricoides***	
Prevalence–uncorrected (95% CI)	29.7 (19.6, 42.3)
Prevalence–corrected (95% BCI)*	31.4 (13.7, 49.4)
Intensity–median epg (range)	336 (48–11376)
Light/Moderate/Heavy intensities (#)**	18/1/0
***T*. *trichiura***	
Prevalence–uncorrected (95% CI)	26.6 (17.0, 39.0)
Prevalence–corrected (95% BCI)*	18.5 (1.0, 43.5)
Intensity–median epg (range)	96 (24–1704)
Light/Moderate/Heavy intensities (#)**	16/1/0
**Hookworm**	
Prevalence–uncorrected (95% CI)	6.3 (2.3, 15.9)
Prevalence–corrected (95% BCI)*	28.7 (1.9, 88.6)
Intensity–median epg (range)	120 (48–168)
Light/Moderate/Heavy intensities (#)**	4/0/0
**Any STH**	
Prevalence–uncorrected (95% CI)	48.4 (36.2, 60.9)

STH = soil-transmitted helminth; epg = eggs per gram (of feces);

CI = confidence interval; BCI = Bayesian credible interval

*Corrected prevalences were calculated using Bayesian estimation of disease prevalence in the absence of a gold standard. A uniform uninformative prior distribution (i.e., beta distribution with parameters α = 1, β = 1) was used for the prevalence; informative information for prior distributions of sensitivity and specificity were taken from Tarafder *et al*. 2010 [28].

** based on WHO intensity categories [40].

During pre-recruitment, 450 participants (44.6%), of the total study population of 1010 participants provided a stool specimen that was stored and analyzed following the 6-month study visit using the direct smear and ethyl-ether concentration techniques. The baseline prevalence of any STH infection was 32.0% (144/450), and the species-specific prevalences of *A*. *lumbricoides*, *T*. *trichiura*, and hookworm infections were 24.7% (111/450), 8.9% (40/450), and 2.7% (12/450), respectively. Prevalences of *T*. *trichiura* and hookworm were similar between intervention groups. A small difference between groups was found in the proportion of participants infected with *A*. *lumbricoides* (experimental group: 29.2%; control group: 20.1%). Because these baseline prevalences, although known to be underestimates due to the limitations of the diagnostic techniques used on the fixed and stored stool specimens, originate from the entire study population, data analyses investigating effects between the intervention groups use these prevalences (the prevalences based on the Kato-Katz technique originating only from the experimental group).

### Follow-up

Follow-up for the 1-month study visit took place between March and August 2014, and follow-up for the 6-month study visit took place between August 2014 and February 2015. A total of 968 (95.8%) infants completed both their 1 and 6-month study visits. Of the 42 infants who were lost-to-follow-up, 7 (0.7%) were visited only at baseline, 31 (3.1%) were visited at 1 month only, and 4 (0.4%) were visited at 6 months only. The causes of attrition were: emigration from study area (n = 35), infant death (n = 5), maternal death (n = 1), and temporary withdrawal (n = 1). The mean number of days between the baseline and first follow-up visit was 32.0 days (±3.6) and the mean number of days between the baseline and second follow-up visit was 186.7 days (±13.3). Follow-up rates were similar in the intervention groups at both follow-up time points. Overall, the majority of baseline characteristics were similar between participants who remained in the trial at 6 months and those who were lost to follow-up (S1 Table). However, infants who missed their 6-month study visit had mothers who were more likely to be single (RR: 2.6; 95% CI: 1.3, 5.3). The proportion of women who reported having received deworming outside of the trial protocol was 3.1% (15/490) in the experimental group and 2.7% (13/478) in the control group. Of the 144 mothers who tested positive for infection with any STH infection (i.e., using the direct smear and ethyl-ether concentration techniques) at baseline, 142 completed their 1-month study visit and 139 completed their 6-month study visit.

### Effect of deworming on primary and secondary anthropometric outcomes

Weight, length and HC gains, as well as z-scores for WAZ, LAZ, HCAZ, and ACAZ between intervention groups at 6 months postpartum are compared in Table 3. The primary outcome, mean infant weight gain between birth and 6 months of age, was similar between intervention groups (4.3 kg ±0.04 *vs*. 4.4 kg ±0.04).

**Table 3:** Effect of maternal postpartum deworming on infant anthropometric outcomes over their first 6 months of life (N = 1010*), Iquitos, Peru (February 2014 –February 2015).

Outcome	Albendazole (n = 510)	Placebo (n = 500)
**Mean weight gain** ±SE (kg), 0–6 mo	4.3±0.04	4.4±0.04
Unadjusted difference (95% CI)	-0.02 (-0.1, 0.08)	*reference*
*p value*	0.675	
Adjusted** difference (95% CI)	-0.01 (-0.1, 0.09)	*reference*
*p value*	0.813	
**Mean length gain** ±SE (cm), 0–6 mo	16.1 ±0.08	16.0 ±0.09
Unadjusted difference (95% CI)	0.08 (-0.2, 0.3)	*reference*
*p value*	0.539	
Adjusted** difference (95% CI)	0.1 (-0.1, 0.3)	*reference*
*p value*	0.398	
**Mean head circumference gain** ±SE (cm), 0–6 mo	8.5 ±0.05	8.5 ±0.05
Unadjusted difference (95% CI)	0.04 (-0.1, 0.2)	*reference*
*p value*	0.612	
Adjusted** difference (95% CI)	0.04 (-0.09, 0.2)	*reference*
*p value*	0.514	
**WAZ** ±SE, 6 mo	-0.2 ±0.05	-0.2 ±0.04
Unadjusted difference (95% CI)	-0.04 (-0.2, 0.09)	*reference*
*p value*	0.551	
Adjusted** difference (95% CI)	-0.05 (-0.2, 0.08)	*reference*
*p value*	0.456	
**WFL** ±SE, 6 mo	0.6 ±0.04	0.6 ±0.05
Unadjusted difference (95% CI)	-0.08 (-0.2, 0.04)	*reference*
*p value*	0.209	
Adjusted** difference (95% CI)	-0.07 (-0.2, 0.05)	*reference*
*p value*	0.257	
**LAZ** ±SE, 6 mo	-1.0 ±0.04	-1.0 ±0.04
Unadjusted difference (95% CI)	0.03 (-0.08, 0.2)	*reference*
*p value*	0.555	
Adjusted** difference (95% CI)	0.01 (-0.1, 0.1)	*reference*
*p value*	0.840	
**HCAZ** ±SE, 6 mo	-0.6 ±0.04	-0.6 ±0.04
Unadjusted difference (95% CI)	-0.02 (-0.1, 0.09)	*reference*
*p value*	0.731	
Adjusted** difference (95% CI)	-0.05 (-0.2, 0.06)	*reference*
*p value*	0.402	
**ACAZ** ±SE, 6 mo	0.1 ±0.04	0.1 ±0.04
Unadjusted difference (95% CI)	0.004 (-0.1, 0.1)	*reference*
*p value*	0.945	
Adjusted** difference (95% CI)	-0.006 (-0.1, 0.09)	*reference*
*p value*	0.912	

SE = standard error (provided by the software Stata/SE version 14.0 for multiple imputation); WAZ = weight-for-age; WFL = weight-for-length; LAZ = length-for-age; HCAZ = head circumference-for-age; ACAZ = mid-upper arm circumference-for-age;

CI = confidence interval

*Intention-to-treat analysis includes data from 972 infants for whom anthropometric outcomes were available, and 38 infants who were lost to follow-up and whose outcome data were imputed using multiple imputation.

**Adjusted for maternal age, education, socioeconomic index, infant sex, and gestational age

There were no differences between groups in terms of the secondary anthropometric outcomes in unadjusted or adjusted ITT analyses (Table 4).

**Table 4:** Effect of maternal postpartum deworming on prevalence of infant underweight, wasting, and stunting at 6 months of age (N = 1010*), Iquitos, Peru (August 2014 –February 2015).

Outcome	Albendazole (n = 510)	Placebo (n = 500)
**Prevalence underweight** (95% CI), 6 mo	3.9 (2.2, 5.6)	3.4 (1.8, 5.0)
Unadjusted RR (95% CI)	1.1 (0.6, 2.2)	*reference*
*p value*	0.691	
Adjusted** RR (95% CI)	1.2 (0.6, 2.3)	*reference*
*p value*	0.584	
**Prevalence wasted** (95% CI), 6 mo	0.8 (0.02, 1.6)	1.2 (0.2, 2.2)
Unadjusted RR (95% CI)	0.7 (0.2, 2.3)	*reference*
*p value*	0.502	
Adjusted** RR (95% CI)	0.6 (0.2, 2.3)	*reference*
*p value*	0.491	
**Prevalence stunted** (95% CI), 6 mo	12.8 (9.8, 15.7)	14.2 (11.1, 17.3)
Unadjusted RR (95% CI)	0.9 (0.7, 1.2)	*reference*
*p value*	0.496	
Adjusted** RR (95% CI)	0.9 (0.7, 1.3)	*reference*
*p value*	0.656	

RR = risk ratio; CI = confidence interval

*Intention-to-treat analysis includes data from 972 infants for whom anthropometric outcomes were available, and 38 infants who were lost to follow-up and whose outcome data were imputed using multiple imputation.

**Adjusted for maternal age, education, socioeconomic index, infant sex, and gestational age

Results were consistent in complete-case analysis (S2 Table and S3 Table) and per-protocol analysis (S4 Table and S5 Table). In *ad hoc* subgroup analyses restricted to the 139 mothers who were found to be positive for any STH infection at baseline (i.e., using the direct smear and ethyl-ether concentration techniques) and who were visited at 6 months postpartum, infants in the experimental group had greater growth in terms of mean length gain in cm (mean difference: 0.8; 95% CI: 0.1, 1.4) and length-for-age in z-score (mean difference: 0.5; 95% CI: 0.2, 0.8) (Table 5), but had similar prevalences of underweight and stunting (S6 Table) at 6 months postpartum.

**Table 5:** Effect of maternal postpartum deworming on infant anthropometric outcomes over their first 6 months of life in women who tested positive for infection with any helminth species at baseline (N = 139*), Iquitos, Peru (February 2014 –February 2015).

Outcome	Albendazole (n = 61)	Placebo (n = 78)
**Mean weight gain** ±SD (kg), 0–6 mo	4.4 ±0.7	4.2 ±0.7
Unadjusted difference (95% CI)	0.2 (-0.005, 0.5)	*reference*
*p value*	0.055	
Adjusted** difference (95% CI)	0.2 (-0.04, 0.5)	*reference*
*p value*	0.099	
**Mean length gain** ±SD (cm), 0–6 mo	16.4 ±1.6	15.6 ±2.2
Unadjusted difference (95% CI)	0.8 (0.1, 1.4)	*reference*
*p value*	0.019	
Adjusted** difference (95% CI)	0.9 (0.3, 1.6)	*reference*
*p value*	0.007	
**Mean head circumference gain** ±SD (cm), 0–6 mo	8.1 ±1.2	8.4 ±1.0
Unadjusted difference (95% CI)	-0.2 (-0.6, 0.2)	*reference*
*p value*	0.265	
Adjusted** difference (95% CI)	-0.2 (-0.6, 0.1)	*reference*
*p value*	0.277	
**WAZ** ±SD, 6 mo	-0.006 ±1.0	-0.4 ±1.0
Unadjusted difference (95% CI)	0.4 (0.09, 0.7)	*reference*
*p value*	0.013	
Adjusted** difference (95% CI)	0.3 (-0.1, 0.6)	*reference*
*p value*	0.061	
**WFL** ±SD, 6 mo	0.7 ±0.9	0.5 ±0.9
Unadjusted difference (95% CI)	0.2 (-0.2, 0.5)	*reference*
*p value*	0.317	
Adjusted** difference (95% CI)	0.08 (-0.2, 0.4)	*reference*
*p value*	0.632	
**LAZ** ±SD, 6 mo	-0.8 ±0.9	-1.2 ±0.9
Unadjusted difference (95% CI)	0.5 (0.2, 0.8)	*reference*
*p value*	0.003	
Adjusted** difference (95% CI)	0.4 (0.09, 0.7)	*reference*
*p value*	0.012	
**HCAZ** ±SD, 6 mo	-0.7 ±0.9	-0.6 ±0.8
Unadjusted difference (95% CI)	-0.03 (-0.3, 0.3)	*reference*
*p value*	0.827	
Adjusted** difference (95% CI)	-0.1 (-0.4, 0.2)	*reference*
*p value*	0.512	
**ACAZ** ±SD, 6 mo	0.2 ±0.8	-0.1 ±0.8
Unadjusted difference (95% CI)	0.3 (0.002, 0.5)	*reference*
*p value*	0.049	
Adjusted** difference (95% CI)	0.2 (-0.1, 0.4)	*reference*
*p value*	0.236	

SD = standard deviation; WAZ = weight-for-age; WFL = weight-for-length; LAZ = length-for-age; HCAZ = head circumference-for-age; ACAZ = mid-upper arm circumference-for-age;

CI = confidence interval

*Analyses restricted to 139 participants who tested positive for infection with any soil-transmitted helminth infection at baseline using either the direct smear or the ethyl-ether concentration techniques and who had outcome data available at 6 months postpartum.

**Adjusted for maternal age, education, socioeconomic index, infant sex, and gestational age

Deworming did not have a statistically significant effect on growth outcomes measured at 1 month postpartum (S7 Table and S8 Table). There was no statistical evidence that the effect of deworming on anthropometric outcomes differed between boys and girls, or varied according to birthweight or birth length.

### Effect of deworming on infant morbidity

The occurrence of hospitalization, and the incidence of diarrhea, respiratory problems, and fever, as assessed at 6 months postpartum are shown in Table 6. The incidence of ear infection did not differ between intervention groups but was too low (< 1%) to perform statistical modelling. No significant difference was observed in indicators of infant morbidity between intervention groups. Results were consistent in complete-case analysis (S9 Table), per-protocol analysis (S10 Table) and *ad hoc* analyses restricted to mothers who tested positive for STH infection at baseline (S11 Table). Moreover, deworming did not have a statistically significant effect on infant morbidity measured at 1 month postpartum (S12 Table).

**Table 6:** Effect of maternal postpartum deworming on infant morbidity indicators at 6 months of age (N = 1010*), Iquitos, Peru (February 2014 –February 2015).

Outcome	Albendazole (n = 510)	Placebo (n = 500)
**Hospitalizations** % (95% CI), 0–6 mo	6.8 (4.6, 9.0)	5.5 (3.5, 7.6)
Unadjusted RR (95% CI)	1.2 (0.7, 2.0)	*reference*
*p value*	0.423	
Adjusted** RR (95% CI)	1.3 (0.8, 2.1)	*reference*
*p value*	0.300	
**Diarrhea** % (95% CI), 6 mo	8.8 (6.3, 11.4)	9.1 (6.5, 11.7)
Unadjusted RR (95% CI)	1.0 (0.6, 1.4)	*reference*
*p value*	0.837	
Adjusted** RR (95% CI)	1.0 (0.6, 1.4)	*reference*
*p value*	0.824	
**Cough** % (95% CI), 6 mo	14.1 (11.1, 17.2)	13.3 (10.2, 16.4)
Unadjusted RR (95% CI)	1.1 (0.8, 1.5)	*reference*
*p value*	0.687	
Adjusted** RR (95% CI)	1.0 (0.8, 1.4)	*reference*
*p value*	0.839	
**Fever** % (95% CI), 6 mo	28.9 (24.9, 32.9)	25.8 (21.8, 29.7)
Unadjusted RR (95% CI)	1.1 (0.9, 1.4)	*reference*
*p value*	0.270	
Adjusted** RR (95% CI)	1.1 (0.9, 1.4)	*reference*
*p value*	0.196	

RR = risk ratio; CI = confidence interval

*Intention-to-treat analysis includes data from 972 infants for whom anthropometric outcomes were available, and 38 infants who were lost to follow-up and whose outcome data were imputed using multiple imputation.

**Adjusted for maternal age, education, socioeconomic index, infant sex, and gestational age

### Serious adverse events

Between baseline and the 6-month study visit, 65 SAEs were reported in infants. The frequency of SAEs was similar between intervention groups, with 34 (6.7%) occurring in the experimental group, and 31 (6.2%) occurring in the control group. Of the 5 infant deaths that were reported over the 6 months of follow-up, 2 occurred in the experimental group and 3 occurred in the control group. No SAE was found to have been related to the administration of albendazole to the mothers.

## Discussion

This is the first trial assessing the effect of maternal postpartum deworming on infant and maternal health outcomes. We were unable to demonstrate an overall effect of maternal postpartum deworming on infant growth or morbidity indicators up to 6 months of age within the total study population. The relatively low prevalence and intensity of STH infection in this population may have reduced the ability of detecting a benefit due to effect dilution (i.e., the trial population comprising both STH-infected and uninfected mothers). When limiting the analyses to mothers who were infected with any one of the STH species at baseline, there was evidence of a benefit in terms of infant growth (i.e., mean length gain, LAZ) at 6 months postpartum. Results were consistent in unadjusted and adjusted analyses.

Although no previous studies of deworming have been conducted in lactating women, trials have been carried out in pregnant populations. There are inconsistencies in study findings among published reports of trials of deworming in second and third trimester pregnant women where infant growth was an outcome measure. In a trial conducted in Uganda between 2003 and 2005, treatment with albendazole in the second and third trimester of pregnancy showed no benefit in terms of mean birthweight or the proportion of infants born with low birthweight [41]. In contrast, in a trial of single-dose mebendazole and daily elemental iron conducted in Peru, a beneficial effect on the proportion of infants born with very low birthweight (<1500 g) was observed [17]. In the present study, the prevalence (< 50%) and intensity of STH infection was low, and therefore closer to the baseline STH infection profile in the previous Uganda trial (68% prevalence) compared to the previous Peru trial (91% prevalence). A recent Cochrane review of published trials on deworming during the second and third trimester of pregnancy found no overall effect on maternal anemia, low birthweight, preterm birth, or perinatal mortality [42]. However, there was important heterogeneity in baseline STH infection and interventions (i.e., combination of anthelminthic drugs and micronutrient supplementation) among included trials that limits the appropriateness of pooling study results. Moreover, subgroup analyses restricted to those women infected with STH infections at baseline were not performed. The low STH prevalence in the current trial may account for the fact that an effect was not observed in the total study population (which included both infected and uninfected mothers), but that effects were demonstrated when the analyses were restricted to STH-infected mothers at baseline.

Our study has several strengths. First is the minimization of measured and unmeasured confounding by external factors achieved through the RCT design. The sample size was large and recruitment of the study population was completed within a period of six months. A high follow-up rate was maintained throughout the trial, minimizing the potential for bias caused by differential loss to follow-up. In-depth training and standardization of anthropometric measurements ensured a high degree of accuracy and precision of outcome ascertainment, thus reducing the potential for measurement error. While deworming medication is readily available for purchase over-the-counter in the study area, compliance with the study protocol was high and non-differential between intervention groups. The consistency of our results between ITT, complete-case, and per-protocol analyses demonstrates the robustness of study findings. Results from the present study may be generalizable to other populations of lactating women in STH-endemic areas with similar prevalence and intensity profiles, and where deliveries in health facilities are actively promoted.

The limitations encountered in the conduct of this trial include a lower than anticipated baseline prevalence of STH (considerably lower than the 91% reported in Larocque *et al* (2006) from the same study area) [23]. This could be the reason why a benefit of deworming in the total study population was not detected, while *ad hoc* subgroup analyses in STH-infected women at baseline demonstrated a positive effect. Collecting stool specimens from women prior to enrolment was challenging and resulted in a less than optimal yield. WHO recommends use of the Kato-Katz technique on fresh stool specimens for the assessment of STH prevalence and intensity. However, due to ethical constraints, specimens collected from those participants randomized to the placebo group could not be immediately analyzed by this technique. The low number of available fresh stool specimens analyzed by the Kato-Katz technique may have affected the accuracy of STH parameter estimation at baseline. The storage of specimens until the end of the 6-month follow-up visit (i.e., > 1 year after collection) likely affected the integrity of specimens and the ability to detect parasites, especially hookworm eggs [43, 44]. Additionally, the direct smear and ethyl-ether concentration techniques have substantially lower test parameters compared to the Kato-Katz technique, and are unable to accurately assess the intensity of infection, thereby not allowing for the classification of morbidity thresholds established by WHO [40]. This likely resulted in some misclassification of baseline STH infection status, and likely also underestimated the effect of deworming in the analyses restricted to STH-positive women at baseline. Lastly, infant morbidity indicators were based on maternal reporting. We have no reason to believe that reporting differed by intervention group, although misclassification in morbidity status may have reduced the observed differences in morbidity outcomes between the two intervention groups.

It has been well established that only individuals infected with STHs will benefit from deworming and, as a result, WHO only recommends the implementation of deworming programs in areas where the prevalence of infection exceeds 20% [14]. This recommendation is based on the fact that infections of moderate/heavy intensity are generally not found at prevalences lower than 20% [45]. Many argue that in order to evaluate the impact of deworming interventions, analyses must be restricted to those who were infected at baseline, and thus could benefit from treatment [30]. However, new trends in ethics guidelines prevent the detection of STH infection at baseline and subsequent randomization of infected individuals to placebo control groups, thereby withholding treatment from those in need. Without accurate individual participant data on baseline infection status, subgroup analyses restricted to infected populations are limited. For these reasons, the standard parallel RCT design may not be adequate to evaluate the effectiveness of deworming. There is a need for developing more novel research designs, such as stepped-wedge RCTs (i.e., cluster RCTs in which cross-over from control to intervention is randomized until all clusters receive the intervention [46]) with careful consideration of secular trends.

Overall, this is the first trial to provide rigorous empirical evidence on the benefits of maternal postpartum deworming. Study findings support WHO recommendations to include lactating women in deworming programs by demonstrating that this strategy is operationally feasible, culturally acceptable, and safe. Future research is needed, not only on the biological mechanisms underpinning the potential link between maternal postpartum deworming and benefits to the infant, but also in study populations having higher prevalences and intensities of STH infections, especially *T*. *trichiura* and hookworm infections which have a direct effect on anemia. The postpartum period is an ideal time to reach women periodically during their reproductive years because they are easily accessible, especially in areas where hospital-based deliveries are promoted, and where deworming can be easily integrated into standard postpartum care. Targeting treatment to the groups at highest risk of STH infection may produce the greatest health impacts.

## Supporting Information

S1 Checklist (DOCX)

S1 Table Baseline characteristics of participants who completed the 6-month visit (n = 972) compared to those who were lost to follow-up at 6 months (n = 38), Iquitos, Peru (February–August 2014). (DOCX)

S2 Table Effect of maternal postpartum deworming on infant anthropometric outcomes over their first 6 months of life, complete-case analysis (n = 972*), Iquitos, Peru (February 2014 –February 2015). (DOCX)

S3 Table Effect of maternal postpartum deworming on prevalence of infant underweight, wasting, and stunting at 6 month of age, complete-case analysis (N = 972*), Iquitos, Peru (August 2014 –February 2015). (DOCX)

S4 Table Effect of maternal postpartum deworming on infant anthropometric outcomes over their first 6 months of life, per-protocol analysis (n = 939*), Iquitos, Peru (February 2014 –February 2015). (DOCX)

S5 Table Effect of maternal postpartum deworming on prevalence of infant underweight, wasting, and stunting at 6 month of age, per-protocol analysis (N = 939*), Iquitos, Peru (August 2014 –February 2015). (DOCX)

S6 Table Effect of maternal postpartum deworming on prevalence of infant underweight and stunting at 6 month of age in women who tested positive for infection with any helminth species at baseline (N = 139*), Iquitos, Peru (August 2014 –February 2015). (DOCX)

S7 Table Effect of maternal postpartum deworming on infant anthropometric outcomes over their first month of life (N = 1010*), Iquitos, Peru (February–September 2014). (DOCX)

S8 Table Effect of maternal postpartum deworming on prevalence of infant underweight, wasting, and stunting at 1 month of age (N = 1010*), Iquitos, Peru (March–September 2014). (DOCX)

S9 Table Effect of maternal postpartum deworming on infant morbidity indicators at 6 months of age, complete-case analysis (N = 972*), Iquitos, Peru (February 2014 –February 2015). (DOCX)

S10 Table Effect of maternal postpartum deworming on infant morbidity indicators at 6 months of age, per-protocol analysis (N = 939*), Iquitos, Peru (February 2014 –February 2015). (DOCX)

S11 Table Effect of maternal postpartum deworming on infant morbidity indicators at 6 months of age in women who tested positive for infection with any helminth species at baseline (N = 139*), Iquitos, Peru (February 2014 –February 2015). (DOCX)

S12 Table Effect of maternal postpartum deworming on infant morbidity indicators at 1 month of age (N = 1010*), Iquitos, Peru, (March–September 2014). (DOCX)
