# Supplementary material for: A Double-Blind Randomized Controlled Trial of Maternal Postpartum Deworming to Improve Infant Weight Gain in the Peruvian Amazon
Source: PLoS Negl Trop Dis. 2017 Jan 5;11(1):e0005098. doi: 10.1371/journal.pntd.0005098 (PMC5215771; doi:10.1371/journal.pntd.0005098)
Supplement: S1 Table — (DOCX) [file pntd.0005098.s002.docx]

S1 Table: Baseline characteristics of participants who completed the 6-month visit (n=972) compared to those who were lost to follow-up at 6 months (n=38), Iquitos, Peru (February – August 2014).

|  | **Completed**  **6-month visit**  **n = 972** | **Did not complete**  **6-month visit**  **n = 38** |
| --- | --- | --- |
| **Maternal characteristics** | | |
| Age (years) ±SD | 25.5 ±6.9 | 23.3 ±6.5 |
| Married or cohabiting n (%) | 862 (88.7) | 28 (73.7) |
| Primigravida n (%) | 248 (25.5) | 13 (34.2) |
| Less than secondary education n (%) | 565 (58.1) | 25 (65.8) |
| Employment outside home n (%) | 115 (11.8) | 3 (7.9) |
| Deworming during pregnancy n (%) | 19 (2.0) | 0 (0) |
| Iron supplementation during pregnancy n (%) | 921 (94.8) | 34 (89.5) |
| Vaginal delivery n (%) | 745 (76.7) | 26 (68.4) |
| **Infant characteristics** | | |
| Male n (%) | 477 (49.1) | 22 (57.9) |
| Birthweight (kg) ±SD | 3.2 ±0.4 | 3.2 ±0.5 |
| Birth length (cm) ±SD | 48.6 ±1.8 | 48.3 ±2.0 |
| Birth head circumference (cm) ±SD | 33.6 ±1.3 | 33.7 ±1.2 |
| Gestational age (weeks) ±SD | 38.7 ±0.8 | 38.5 ±1.1 |
| Apgar score at 5 minutes | 9.8 ±0.5 | 9.7 ±0.7 |
| **Household characteristics** | | |
| Peri-urban or rural residence n (%) | 937 (96.4) | 38 (100.0) |
| Access to potable water in home n (%) | 737 (75.8) | 26 (68.4) |
| Home with dirt or wooden floor n (%) | 647 (66.6) | 26 (68.4) |
| Number of people residing in household ±SD | 5.8 ±2.7 | 6.1 ±2.7 |

Results expressed as means ±SD or frequency n (%)
