# Supplementary material for: A Double-Blind Randomized Controlled Trial of Maternal Postpartum Deworming to Improve Infant Weight Gain in the Peruvian Amazon
Source: PLoS Negl Trop Dis. 2017 Jan 5;11(1):e0005098. doi: 10.1371/journal.pntd.0005098 (PMC5215771; doi:10.1371/journal.pntd.0005098)
Supplement: S2 Table — (DOCX) [file pntd.0005098.s003.docx]

S2 Table. Effect of maternal postpartum deworming on infant anthropometric outcomes over their first 6 months of life, complete-case analysis (n=972*), Iquitos, Peru (February 2014 – February 2015).

| **Outcome** | **Albendazole**  **n=491** | **Placebo**  **n=481** |
| --- | --- | --- |
| **Mean weight gain** ±SD (kg), 0 – 6 mo | 4.3 ±0.8 | 4.3 ±0.8 |
| Unadjusted difference (95% CI) | -0.02 (-0.1, 0.08) | *reference* |
| *p value* | 0.756 |  |
| Adjusted** difference (95 % CI) | -0.009 (-0.1, 0.09) | *reference* |
| *p value* | 0.857 |  |
| **Mean length gain** ±SD (cm), 0 – 6 mo | 16.1 ±1.9 | 16.0 ±1.9 |
| Unadjusted difference (95% CI) | 0.09 (-0.1, 0.3) | *reference* |
| *p value* | 0.443 |  |
| Adjusted** difference (95 % CI) | 0.1 (-0.1, 0.3) | *reference* |
| p value | 0.349 |  |
| **Mean head circumference gain** ±SD (cm), 0 – 6 mo | 8.5 ±1.1 | 8.5 ±1.1 |
| Unadjusted difference (95% CI) | 0.03 (-0.1, 0.2) | *reference* |
| *p value* | 0.647 |  |
| Adjusted** difference (95 % CI) | 0.04 (-0.1, 0.2) | *reference* |
| *p value* | 0.599 |  |
| **WAZ** ±SD, 6 mo | -0.2 ±1.0 | -0.2 ±1.0 |
| Unadjusted difference (95% CI) | -0.03 (-0.2, 0.09) | *reference* |
| *p value* | 0.620 |  |
| Adjusted** difference (95 % CI) | -0.04 (-0.2, 0.08) | *reference* |
| p value | 0.512 |  |
| **WFL** ±SD, 6 mo | 0.6 ±1.0 | 0.6 ±1.0 |
| Unadjusted difference (95% CI) | -0.08 (-0.2, 0.04) | *reference* |
| *p value* | 0.206 |  |
| Adjusted** difference (95 % CI) | -0.07 (-0.2, 0.05) | *reference* |
| *p value* | 0.251 |  |
| **LAZ** ±SD, 6 mo | -1.0 ±0.9 | -1.0 ±0.9 |
| Unadjusted difference (95% CI) | 0.05 (-0.07, 0.2) | *reference* |
| *p value* | 0.445 |  |
| Adjusted** difference (95 % CI) | 0.02 (-0.09, 0.1) | *reference* |
| p value | 0.706 |  |
| **HCAZ** ±SD, 6 mo | -0.6 ±0.9 | -0.6 ±0.9 |
| Unadjusted difference (95% CI) | -0.01 (-0.1, 0.1) | *reference* |
| *p value* | 0.824 |  |
| Adjusted** difference (95 % CI) | -0.04 (-0.1, 0.07) | *reference* |
| *p value* | 0.465 |  |
| **ACAZ** ±SD, 6 mo | 0.1 ±0.8 | 0.1 ±0.8 |
| Unadjusted difference (95% CI) | 0.003 (-0.1, 0.1) | *reference* |
| *p value* | 0.953 |  |
| Adjusted** difference (95 % CI) | -0.006 (-0.1, 0.1) | *reference* |
| *p value* | 0.908 |  |

SD= standard deviation; WAZ= weight-for-age; WFL= weight-for-length; LAZ= length-for-age; HCAZ= head circumference-for-age; ACAZ= mid-upper arm circumference-for-age;

CI= confidence interval

*Complete-case analysis includes data from 972 infants for whom anthropometric outcomes were available at 6 months postpartum.

**Adjusted for maternal age, education, socioeconomic index, infant sex, and gestational age
