# Supplementary material for: A Double-Blind Randomized Controlled Trial of Maternal Postpartum Deworming to Improve Infant Weight Gain in the Peruvian Amazon
Source: PLoS Negl Trop Dis. 2017 Jan 5;11(1):e0005098. doi: 10.1371/journal.pntd.0005098 (PMC5215771; doi:10.1371/journal.pntd.0005098)
Supplement: S3 Table — (DOCX) [file pntd.0005098.s004.docx]

S3 Table. Effect of maternal postpartum deworming on prevalence of infant underweight, wasting, and stunting at 6 month of age, complete-case analysis (N=972*), Iquitos, Peru (August 2014 – February 2015).

| **Outcome** | **Albendazole**  **n=491** | **Placebo**  **n=481** |
| --- | --- | --- |
| **Prevalence underweight** (95% CI), 6 mo | 3.9 (2.5, 6.0) | 3.3 (2.0, 5.4) |
| Unadjusted RR (95% CI) | 1.2 (0.6, 2.2) | *reference* |
| *p value* | 0.650 |  |
| Adjusted** RR (95 % CI) | 1.2 (0.6, 2.3) | *reference* |
| *p value* | 0.548 |  |
| **Prevalence wasted** (95% CI), 6 mo | 0.8 (0.3, 2.2) | 1.2 (0.6, 2.8) |
| Unadjusted RR (95% CI) | 0.7 (0.2, 2.3) | *reference* |
| *p value* | 0.507 |  |
| Adjusted† RR (95 % CI) | 0.7 (0.2, 2.3) | *reference* |
| *p value* | 0.519 |  |
| **Prevalence stunted** (95% CI), 6 mo | 12.6 (10.0, 15.9) | 14.1 (11.3, 17.6) |
| Unadjusted RR (95% CI) | 0.9 (0.6, 1.2) | *reference* |
| *p value* | 0.490 |  |
| Adjusted** RR (95 % CI) | 0.9 (0.7, 1.3) | *reference* |
| *p value* | 0.630 |  |

RR= risk ratio; CI= confidence interval

*Complete-case analysis includes data from 972 infants for whom anthropometric outcomes were available at 6 months postpartum.

**Adjusted for maternal age, education, socioeconomic index, infant sex, and gestational age

†Adjusted for maternal age, education, infant sex, and gestational age (socioeconomic index variable removed because of spare data)
