# Supplementary material for: A Double-Blind Randomized Controlled Trial of Maternal Postpartum Deworming to Improve Infant Weight Gain in the Peruvian Amazon
Source: PLoS Negl Trop Dis. 2017 Jan 5;11(1):e0005098. doi: 10.1371/journal.pntd.0005098 (PMC5215771; doi:10.1371/journal.pntd.0005098)
Supplement: S4 Table — (DOCX) [file pntd.0005098.s005.docx]

S4 Table. Effect of maternal postpartum deworming on infant anthropometric outcomes over their first 6 months of life, per-protocol analysis (n=939*), Iquitos, Peru (February 2014 – February 2015).

| **Outcome** | **Albendazole**  **n=475** | **Placebo**  **n=464** |
| --- | --- | --- |
| **Mean weight gain** ±SD (kg), 0 – 6 mo | 4.3 ±0.8 | 4.3 ±0.8 |
| Unadjusted difference (95% CI) | -0.02 (-0.1, 0.09) | *reference* |
| *p value* | 0.740 |  |
| Adjusted** difference (95 % CI) | -0.01 (-0.1, 0.09) | *reference* |
| *p value* | 0.832 |  |
| **Mean length gain** ±SD (cm), 0 – 6 mo | 16.1 ±1.9 | 16.0 ±2.0 |
| Unadjusted difference (95% CI) | 0.1 (-0.1, 0.3) | *reference* |
| *p value* | 0.433 |  |
| Adjusted** difference (95 % CI) | 0.1 (-0.1, 0.4) | *reference* |
| *p value* | 0.338 |  |
| **Mean head circumference gain** ±SD (cm), 0 – 6 mo | 8.5 ±1.1 | 8.5 ±1.1 |
| Unadjusted difference (95% CI) | 0.02 (-0.1, 0.2) | *reference* |
| *p value* | 0.739 |  |
| Adjusted** difference (95 % CI) | 0.03 (-0.1, 0.2) | *reference* |
| *p value* | 0.680 |  |
| **WAZ** ±SD, 6 mo | -0.2 ±1.0 | -0.2 ±1.0 |
| Unadjusted difference (95% CI) | -0.03 (-0.2, 0.1) | *reference* |
| *p value* | 0.673 |  |
| Adjusted** difference (95 % CI) | -0.04 (-0.2, 0.09) | *reference* |
| *p value* | 0.575 |  |
| **WFL** ±SD, 6 mo | 0.6 ±1.0 | 0.6 ±1.0 |
| Unadjusted difference (95% CI) | -0.08 (-0.2, 0.05) | *reference* |
| *p value* | 0.219 |  |
| Adjusted** difference (95 % CI) | -0.07 (-0.2, 0.06) | *reference* |
| *p value* | 0.267 |  |
| **LAZ** ±SD, 6 mo | -1.0 ±0.9 | -1.0 ±0.9 |
| Unadjusted difference (95% CI) | 0.05 (-0.07, 0.2) | *reference* |
| *p value* | 0.398 |  |
| Adjusted** difference (95 % CI) | 0.03 (-0.09, 0.1) | *reference* |
| *p value* | 0.621 |  |
| **HCAZ** ±SD, 6 mo | -0.6 ±0.9 | -0.6 ±0.9 |
| Unadjusted difference (95% CI) | -0.01 (-0.1, 0.1) | *reference* |
| *p value* | 0.819 |  |
| Adjusted** difference (95 % CI) | -0.04 (-0.1, 0.07) | *reference* |
| *p value* | 0.494 |  |
| **ACAZ** ±SD, 6 mo | 0.2 ±0.8 | 0.1 ±0.8 |
| Unadjusted difference (95% CI) | 0.007 (-0.1, 0.1) | *reference* |
| *p value* | 0.904 |  |
| Adjusted** difference (95 % CI) | -0.0008 (-0.1, 0.1) | *reference* |
| *p value* | 0.988 |  |

SD= standard deviation; WAZ= weight-for-age; WFL= weight-for-length; LAZ= length-for-age; HCAZ= head circumference-for-age; ACAZ= mid-upper arm circumference-for-age; CI= confidence interval

*Per-protocol analysis includes data from 939 infants for whom anthropometric outcomes were available at 1 and 6 months postpartum, and whose mothers did not report taking deworming outside of the trial protocol

**Adjusted for maternal age, education, socioeconomic index, infant sex, and gestational age
