# Supplementary material for: A Double-Blind Randomized Controlled Trial of Maternal Postpartum Deworming to Improve Infant Weight Gain in the Peruvian Amazon
Source: PLoS Negl Trop Dis. 2017 Jan 5;11(1):e0005098. doi: 10.1371/journal.pntd.0005098 (PMC5215771; doi:10.1371/journal.pntd.0005098)
Supplement: S5 Table — (DOCX) [file pntd.0005098.s006.docx]

S5 Table. Effect of maternal postpartum deworming on prevalence of infant underweight, wasting, and stunting at 6 month of age, per-protocol analysis (N=939*), Iquitos, Peru (August 2014 – February 2015).

| **Outcome** | **Albendazole**  **n=475** | **Placebo**  **n=464** |
| --- | --- | --- |
| **Prevalence underweight** (95% CI), 6 mo | 4.0 (2.6, 6.2) | 3.4 (2.1, 5.6) |
| Unadjusted RR (95% CI) | 1.2 (0.6, 2.2) | *reference* |
| *p value* | 0.656 |  |
| Adjusted** RR (95 % CI) | 1.2 (0.6, 2.3) | *reference* |
| *p value* | 0.560 |  |
| **Prevalence wasted** (95% CI), 6 mo | 0.8 (0.3, 2.2) | 1.3 (0.6, 2.9) |
| Unadjusted RR (95% CI) | 0.7 (0.2, 2.3) | *reference* |
| *p value* | 0.504 |  |
| Adjusted† RR (95 % CI) | 0.7 (0.2, 2.3) | *reference* |
| *p value* | 0.512 |  |
| **Prevalence stunted** (95% CI), 6 mo | 12.6 (9.9, 15.9) | 14.0 (11.1, 17.5) |
| Unadjusted RR (95% CI) | 0.9 (0.7, 1.3) | *reference* |
| *p value* | 0.535 |  |
| Adjusted** RR (95 % CI) | 0.9 (0.7, 1.3) | *reference* |
| *p value* | 0.684 |  |

RR= risk ratio; CI= confidence interval

*Per-protocol analysis includes data from 939 infants for whom anthropometric outcomes were available at 1 and 6 months postpartum, and whose mothers did not report taking deworming outside of the trial protocol

**Adjusted for maternal age, education, socioeconomic index, infant sex, and gestational age

†Adjusted for maternal age, education, infant sex, and gestational age (socioeconomic index variable removed because of spare data)
