# Supplementary material for: A Double-Blind Randomized Controlled Trial of Maternal Postpartum Deworming to Improve Infant Weight Gain in the Peruvian Amazon
Source: PLoS Negl Trop Dis. 2017 Jan 5;11(1):e0005098. doi: 10.1371/journal.pntd.0005098 (PMC5215771; doi:10.1371/journal.pntd.0005098)
Supplement: S6 Table — (DOCX) [file pntd.0005098.s007.docx]

S6 Table. Effect of maternal postpartum deworming on prevalence of infant underweight and stunting at 6 month of age in women who tested positive for infection with any helminth species at baseline (N=139*), Iquitos, Peru (August 2014 – February 2015).

| **Outcome**† | **Albendazole**  **n=61** | **Placebo**  **n=78** |
| --- | --- | --- |
| **Prevalence underweight** (95% CI), 6 mo | 1.6 (0.2, 11.3) | 3.8 (0.1, 11.5) |
| Unadjusted RR (95% CI) | 0.4 (0.05, 4.0) | *reference* |
| *p value* | 0.455 |  |
| Adjusted** RR (95 % CI) | 0.6 (0.07, 5.9) | *reference* |
| *p value* | 0.682 |  |
| **Prevalence stunted** (95% CI), 6 mo | 11.5 (5.4, 22.6) | 16.7 (9.8, 26.9) |
| Unadjusted RR (95% CI) | 0.7 (0.3, 1.6) | *reference* |
| *p value* | 0.393 |  |
| Adjusted** RR (95 % CI) | 0.8 (0.4, 1.9) | *reference* |
| *p value* | 0.653 |  |

RR= risk ratio; CI= confidence interval

*Analyses restricted to 139 participants who tested positive for infection with any soil-transmitted helminth infection at baseline using either the direct smear or the ethyl-ether concentration techniques and who had outcome data available at 6 months postpartum.

**Adjusted for maternal age, education, infant sex, and gestational age (socioeconomic index variable removed because of spare data)

†Prevalence wasted is not shown because the prevalence was too low (<1%) for estimation
