# Supplementary material for: A Double-Blind Randomized Controlled Trial of Maternal Postpartum Deworming to Improve Infant Weight Gain in the Peruvian Amazon
Source: PLoS Negl Trop Dis. 2017 Jan 5;11(1):e0005098. doi: 10.1371/journal.pntd.0005098 (PMC5215771; doi:10.1371/journal.pntd.0005098)
Supplement: S7 Table — (DOCX) [file pntd.0005098.s008.docx]

S7 Table. Effect of maternal postpartum deworming on infant anthropometric outcomes over their first month of life (N=1010*), Iquitos, Peru (February – September 2014).

| **Outcome** | **Albendazole**  **n=510** | **Placebo**  **n=500** |
| --- | --- | --- |
| **Mean weight gain** ±SE (kg), 0 – 1 mo | 1.0 ±0.02 | 1.0 ±0.02 |
| Unadjusted difference (95% CI) | -0.02 (-0.07, 0.03) | *reference* |
| *p value* | 0.416 |  |
| Adjusted** difference (95 % CI) | -0.02 (-0.07, 0.03) | *reference* |
| *p value* | 0.379 |  |
| **Mean length gain** ±SE (cm), 0 – 1 mo | 4.0 ±0.06 | 4.0 ±0.06 |
| Unadjusted difference (95% CI) | -0.01 (-0.2, 0.2) | *reference* |
| *p value* | 0.892 |  |
| Adjusted** difference (95 % CI) | 0.009 (-0.2, 0.2) | *reference* |
| *p value* | 0.917 |  |
| **Mean head circumference gain** ±SE (cm), 0 – 1 mo | 3.1 ±0.04 | 3.1 ±0.04 |
| Unadjusted difference (95% CI) | 0.04 (-0.07, 0.2) | *reference* |
| *p value* | 0.461 |  |
| Adjusted** difference (95 % CI) | 0.04 (-0.07, 0.1) | *reference* |
| *p value* | 0.468 |  |
| **WAZ** ±SE, 1 mo | -0.5 ±0.05 | -0.4 ±0.04 |
| Unadjusted difference (95% CI) | -0.07 (-0.2, 0.05) | *reference* |
| *p value* | 0.274 |  |
| Adjusted** difference (95 % CI) | -0.09 (-0.2, 0.02) | *reference* |
| *p value* | 0.112 |  |
| **WFL** ±SE, 1 mo | 0.5 ±0.05 | 0.6 ±0.04 |
| Unadjusted difference (95% CI) | -0.1 (-0.2, 0.01) | *reference* |
| *p value* | 0.085 |  |
| Adjusted** difference (95 % CI) | -0.1 (-0.2, -0.006) | *reference* |
| *p value* | 0.063 |  |
| **LAZ** ±SE, 1 mo | -0.9 ±0.04 | -0.9 ±0.04 |
| Unadjusted difference (95% CI) | 0.0006 (-0.1, 0.1) | *reference* |
| *p value* | 0.991 |  |
| Adjusted** difference (95 % CI) | -0.02 (-0.1, 0.09) | *reference* |
| *p value* | 0.709 |  |
| **HCAZ** ±SE, 1 mo | -0.3 ±0.04 | -0.3 ±0.04 |
| Unadjusted difference (95% CI) | -0.01 (-0.1, 0.09) | *reference* |
| *p value* | 0.792 |  |
| Adjusted** difference (95 % CI) | -0.05 (-0.2, 0.06) | *reference* |
| *p value* | 0.391 |  |

SE = standard error (provided by the software Stata/SE version 14.0 for multiple imputation); WAZ= weight-for-age; WFL= weight-for-length; LAZ= length-for-age; HCAZ= head circumference-for-age; CI= confidence interval

*Intention-to-treat analysis includes data from 999 infants for whom anthropometric outcomes were available, and 11 infants who were lost to follow-up and whose outcome data were imputed using multiple imputation.

**Adjusted for maternal age, education, socioeconomic index, infant sex, and gestational age
