# Supplementary material for: A Double-Blind Randomized Controlled Trial of Maternal Postpartum Deworming to Improve Infant Weight Gain in the Peruvian Amazon
Source: PLoS Negl Trop Dis. 2017 Jan 5;11(1):e0005098. doi: 10.1371/journal.pntd.0005098 (PMC5215771; doi:10.1371/journal.pntd.0005098)
Supplement: S8 Table — (DOCX) [file pntd.0005098.s009.docx]

S8 Table. Effect of maternal postpartum deworming on prevalence of infant underweight, wasting, and stunting at 1 month of age (N=1010*), Iquitos, Peru (March – September 2014).

| **Outcome** | **Albendazole**  **n=510** | **Placebo**  **n=500** |
| --- | --- | --- |
| **Prevalence underweight** (95% CI), 1 mo | 8.7 (6.2, 11.1) | 6.1 (4.0, 8.2) |
| Unadjusted RR (95% CI) | 1.4 (0.9, 2.2) | *reference* |
| *p value* | 0.121 |  |
| Adjusted** RR (95 % CI) | 1.5 (0.9, 2.3) | *reference* |
| *p value* | 0.084 |  |
| **Prevalence wasted** (95% CI), 1 mo | 2.0 (0.8, 3.2) | 1.0 (0.1, 1.9) |
| Unadjusted RR (95% CI) | 1.9 (0.7, 5.6) | *reference* |
| *p value* | 0.229 |  |
| Adjusted** RR (95 % CI) | 1.9 (0.7, 5.6) | *reference* |
| *p value* | 0.235 |  |
| **Prevalence stunted** (95% CI), 1 mo | 11.9 (9.0, 14.7) | 12.9 (9.9, 15.8) |
| Unadjusted RR (95% CI) | 0.9 (0.7, 1.3) | *reference* |
| *p value* | 0.639 |  |
| Adjusted** RR (95 % CI) | 1.0 (0.7, 1.3) | *reference* |
| *p value* | 0.880 |  |

RR= risk ratio; CI= confidence interval

*Intention-to-treat analysis includes data from 999 infants for whom anthropometric outcomes were available, and 11 infants who were lost to follow-up and whose outcome data were imputed using multiple imputation.

**Adjusted for maternal age, education, socioeconomic index, infant sex, and gestational age
