# Supplementary material for: A Double-Blind Randomized Controlled Trial of Maternal Postpartum Deworming to Improve Infant Weight Gain in the Peruvian Amazon
Source: PLoS Negl Trop Dis. 2017 Jan 5;11(1):e0005098. doi: 10.1371/journal.pntd.0005098 (PMC5215771; doi:10.1371/journal.pntd.0005098)
Supplement: S9 Table — (DOCX) [file pntd.0005098.s010.docx]

S9 Table. Effect of maternal postpartum deworming on infant morbidity indicators at 6 months of age, complete-case analysis (N=972*), Iquitos, Peru (February 2014 – February 2015).

| **Outcome** | **Albendazole**  **n=491** | **Placebo**  **n=481** |
| --- | --- | --- |
| **Hospitalizations** % (95% CI), 0 – 6 mo | 6.5 (4.6, 9.1) | 5.4 (3.7, 7.8) |
| Unadjusted RR (95% CI) | 1.2 (0.7, 2.0) | *reference* |
| *p value* | 0.465 |  |
| Adjusted** RR (95 % CI) | 1.3 (0.8, 2.1) | *reference* |
| *p value* | 0.343 |  |
| **Diarrhea** % (95% CI), 6 mo | 8.6 (6.4, 11.4) | 8.9 (6.7, 11.9) |
| Unadjusted RR (95% CI) | 1.0 (0.6, 1.4) | *reference* |
| *p value* | 0.831 |  |
| Adjusted** RR (95 % CI) | 1.0 (0.6, 1.4) | *reference* |
| *p value* | 0.813 |  |
| **Cough** % (95% CI), 6 mo | 14.1 (11.2, 17.4) | 13.3 (10.5, 16.7) |
| Unadjusted RR (95% CI) | 1.1 (0.8, 1.4) | *reference* |
| *p value* | 0.735 |  |
| Adjusted** RR (95 % CI) | 1.0 (0.7, 1.4) | *reference* |
| *p value* | 0.901 |  |
| **Fever** % (95% CI), 6 mo | 28.9 (25.1, 33.1) | 25.8 (22.1, 29.9) |
| Unadjusted RR (95% CI) | 1.1 (0.9, 1.4) | *reference* |
| *p value* | 0.273 |  |
| Adjusted** RR (95 % CI) | 1.1 (0.9, 1.4) | *reference* |
| *p value* | 0.205 |  |

RR= risk ratio; CI= confidence interval

*Complete-case analysis includes data from 972 infants for whom morbidity outcomes were available at 6 months postpartum.

**Adjusted for maternal age, education, socioeconomic index, infant sex, and gestational age
