# Supplementary material for: A Double-Blind Randomized Controlled Trial of Maternal Postpartum Deworming to Improve Infant Weight Gain in the Peruvian Amazon
Source: PLoS Negl Trop Dis. 2017 Jan 5;11(1):e0005098. doi: 10.1371/journal.pntd.0005098 (PMC5215771; doi:10.1371/journal.pntd.0005098)
Supplement: S10 Table — (DOCX) [file pntd.0005098.s011.docx]

S10 Table. Effect of maternal postpartum deworming on infant morbidity indicators at 6 months of age, per-protocol analysis (N=939*), Iquitos, Peru (February 2014 – February 2015).

| **Outcome** | **Albendazole**  **n=475** | **Placebo**  **n=464** |
| --- | --- | --- |
| **Hospitalizations** % (95% CI), 0 – 6 mo | 6.5 (4.6, 9.1) | 5.4 (3.7, 7.9) |
| Unadjusted RR (95% CI) | 1.2 (0.7, 2.0) | *reference* |
| *p value* | 0.462 |  |
| Adjusted** RR (95 % CI) | 1.3 (0.8, 2.1) | *reference* |
| *p value* | 0.357 |  |
| **Diarrhea** % (95% CI), 6 mo | 8.4 (6.2, 11.3) | 9.3 (6.9, 12.3) |
| Unadjusted RR (95% CI) | 0.9 (0.6, 1.4) | *reference* |
| *p value* | 0.648 |  |
| Adjusted** RR (95 % CI) | 0.9 (0.6, 1.4) | *reference* |
| *p value* | 0.652 |  |
| **Cough** % (95% CI), 6 mo | 13.9 (11.1, 17.3) | 13.4 (10.5, 16.8) |
| Unadjusted RR (95% CI) | 1.0 (0.8, 1.4) | *reference* |
| *p value* | 0.812 |  |
| Adjusted** RR (95 % CI) | 1.0 (0.7, 1.4) | *reference* |
| *p value* | 0.944 |  |
| **Fever** % (95% CI), 6 mo | 29.1 (25.1, 33.3) | 25.2 (21.5, 29.4) |
| Unadjusted RR (95% CI) | 1.2 (0.9, 1.4) | *reference* |
| *p value* | 0.187 |  |
| Adjusted** RR (95 % CI) | 1.2 (0.9, 1.4) | *reference* |
| *p value* | 0.146 |  |

RR= risk ratio; CI= confidence interval

*Per-protocol analysis includes data from 939 infants for whom morbidity outcomes were available at 1 and 6 months postpartum, and whose mothers did not report taking deworming outside of the trial protocol

**Adjusted for maternal age, education, socioeconomic index, infant sex, and gestational age
