# Supplementary material for: A Double-Blind Randomized Controlled Trial of Maternal Postpartum Deworming to Improve Infant Weight Gain in the Peruvian Amazon
Source: PLoS Negl Trop Dis. 2017 Jan 5;11(1):e0005098. doi: 10.1371/journal.pntd.0005098 (PMC5215771; doi:10.1371/journal.pntd.0005098)
Supplement: S11 Table — (DOCX) [file pntd.0005098.s012.docx]

S11 Table. Effect of maternal postpartum deworming on infant morbidity indicators at 6 months of age in women who tested positive for infection with any helminth species at baseline (N=139*), Iquitos, Peru (February 2014 – February 2015).

| **Outcome** | **Albendazole**  **n=61** | **Placebo**  **n=78** |
| --- | --- | --- |
| **Hospitalizations** % (95% CI), 0 – 6 mo | 11.5 (5.4, 22.6) | 3.8 (1.2, 11.5) |
| Unadjusted RR (95% CI) | 3.0 (0.8, 11.1) | *reference* |
| *p value* | 0.102 |  |
| Adjusted** RR (95 % CI) | 3.8 (1.0, 14.6) | *reference* |
| *p value* | 0.055 |  |
| **Diarrhea** % (95% CI), 6 mo | 9.8 (4.4, 20.6) | 9.0 (4.3, 17.9) |
| Unadjusted RR (95% CI) | 1.1 (0.4, 3.1) | *reference* |
| *p value* | 0.863 |  |
| Adjusted** RR (95 % CI) | 0.9 (0.3, 2.8) | *reference* |
| *p value* | 0.880 |  |
| **Cough** % (95% CI), 6 mo | 13.1 (6.6, 24.5) | 16.7 (9.8, 26.9) |
| Unadjusted RR (95% CI) | 0.8 (0.3, 1.8) | *reference* |
| *p value* | 0.564 |  |
| Adjusted** RR (95 % CI) | 0.7 (0.3, 1.6) | *reference* |
| *p value* | 0.363 |  |
| **Fever** % (95% CI), 6 mo | 23.0 (13.9, 35.5) | 25.6 (17.0, 36.7) |
| Unadjusted RR (95% CI) | 0.9 (0.5, 1.6) | *reference* |
| *p value* | 0.715 |  |
| Adjusted** RR (95 % CI) | 0.8 (0.4, 1.5) | *reference* |
| *p value* | 0.450 |  |

RR= risk ratio; CI= confidence interval

*Analyses restricted to 139 participants who tested positive for infection with any soil-transmitted helminth infection at baseline using either the direct smear or the ethyl-ether concentration techniques and who had outcome data available at 6 months postpartum.

**Adjusted for maternal age, education, socioeconomic index, infant sex, and gestational age
