# Supplementary material for: A Double-Blind Randomized Controlled Trial of Maternal Postpartum Deworming to Improve Infant Weight Gain in the Peruvian Amazon
Source: PLoS Negl Trop Dis. 2017 Jan 5;11(1):e0005098. doi: 10.1371/journal.pntd.0005098 (PMC5215771; doi:10.1371/journal.pntd.0005098)
Supplement: S12 Table — (DOCX) [file pntd.0005098.s013.docx]

S12 Table. Effect of maternal postpartum deworming on infant morbidity indicators at 1 month of age (N=1010*), Iquitos, Peru, (March – September 2014).

| **Outcome** | **Albendazole**  **n=510** | **Placebo**  **n=500** |
| --- | --- | --- |
| **Diarrhea** % (95% CI), 1 mo | 3.2 (1.6, 4.7) | 2.7 (1.3, 4.1) |
| Unadjusted RR (95% CI) | 1.2 (0.6, 2.4) | *reference* |
| *p value* | 0.644 |  |
| Adjusted** RR (95 % CI) | 1.3 (0.6, 2.6) | *reference* |
| *p value* | 0.482 |  |
| **Cough** % (95% CI), 1 mo | 9.3 (6.7, 11.8) | 7.9 (5.5, 10.3) |
| Unadjusted RR (95% CI) | 1.2 (0.8, 1.8) | *reference* |
| *p value* | 0.447 |  |
| Adjusted** RR (95 % CI) | 1.2 (0.8, 1.8) | *reference* |
| *p value* | 0.377 |  |
| **Fever** % (95% CI), 1 mo | 14.1 (11.0, 17.1) | 13.1 (10.0, 16.1) |
| Unadjusted RR (95% CI) | 1.1 (0.8, 1.5) | *reference* |
| *p value* | 0.647 |  |
| Adjusted** RR (95 % CI) | 1.1 (0.8, 1.6) | *reference* |
| *p value* | 0.447 |  |

RR= risk ratio; CI= confidence interval

*Intention-to-treat analysis includes data from 999 infants for whom morbidity outcomes were available, and 11 infants who were lost to follow-up and whose outcome data were imputed using multiple imputation.

**Adjusted for maternal age, education, socioeconomic index, infant sex, and gestational age
